# Exosome-Mediated Intercellular Communication in the Pathological Processes of Gouty Arthritis and Its Treatment

**DOI:** 10.3390/ijms27041656

**Published:** 2026-02-08

**Authors:** Wenren Zhao, Wenhao Zhong, Zexuan Wang, Qian Zhou, Yu Wang, Bing Zhang, Zhijian Lin

**Affiliations:** 1 Department of Clinical Chinese Pharmacy, School of Chinese Pharmacy, Beijing University of Chinese Medicine, Beijing 100029, China; zhaowenren22@163.com (W.Z.); zhongwenhao233@163.com (W.Z.); 17736275905@163.com (Z.W.); 18375069361@163.com (Q.Z.); wangyuxh@163.com (Y.W.); zhangb@bucm.edu.cn (B.Z.); 2 Research Center for Pharmacovigilance and Rational Use of Chinese Medicine, Beijing University of Chinese Medicine, Beijing 100029, China

**Keywords:** gouty arthritis, exosomes, intercellular communication, monosodium urate, NLRP3 inflammasome, microRNA, therapeutic targeting, complement system, immunomodulation

## Abstract

Gouty arthritis (GA) is a debilitating autoinflammatory disorder precipitated by the deposition of monosodium urate (MSU) crystals, leading to intense, recurrent joint inflammation and systemic metabolic dysregulation. While hyperuricemia is a prerequisite, the transition to clinical gout involves complex intercellular signaling cascades that are not fully understood. Emerging evidence has identified exosomes,— nanoscale extracellular vesicles, —as critical mediators in this pathological process. Exosomes function as intercellular carriers, transporting a diverse cargo of bioactive molecules, including proteins, lipids, and nucleic acids (e.g., microRNAs), which profoundly influence immune cell activation, inflammasome regulation, and metabolic pathways. This review provides a critical analysis of the dual role of exosomes in both propagating and potentially resolving inflammation in GA. We delve into the intricate mechanisms of exosome-mediated pathogenesis, including the modulation of purine metabolism, lysosomal function, and complement–inflammasome crosstalk. Furthermore, we explore the burgeoning field of exosome-based therapeutics, critically evaluating strategies such as engineered exosomes for targeted drug delivery, mesenchymal stem cell (MSC)-derived exosomes for immunomodulation, and the development of exosomal biomarkers for diagnostics. Additionally, we examine how chemical drugs and herbal compounds may exert therapeutic effects by modulating exosome pathways, offering new insights into integrative treatment approaches. By synthesizing recent findings from proteomic, transcriptomic, and functional studies, we aim to unravel the complexities of exosome signaling in GA and to propose innovative therapeutic avenues that target these pathways to improve patient outcomes.

## 1. Introduction

Gouty arthritis, one of the most prevalent forms of inflammatory arthritis, is characterized by recurrent episodes of acute inflammation due to monosodium urate (MSU) crystal deposition in the joints and surrounding tissues [1]. MSU crystals primarily deposit in the joints and periarticular tissues, where they are recognized by resident macrophages and other innate immune cells via surface receptors (e.g., Toll-like receptors) and by direct phagocytosis. This interaction triggers the initial innate immune signaling cascade, culminating in NLRP3 inflammasome activation and IL-1β release, which drives the acute inflammatory response characteristic of GA [2]. The pathophysiology of gout is complex and primarily involves chronic hyperuricemia, which results from increased uric acid production and/or decreased renal excretion of uric acid [3,4]. Hyperuricemia leads to the formation of MSU crystals, triggering an innate immune response mediated by the nucleotide-binding oligomerization domain-, leucine-rich repeat-, and pyrin domain-containing protein 3 (NLRP3) inflammasome [5]. Activation of NLRP3 results in the cleavage of pro-interleukin-1β (IL-1β) into its active form, which is a key pro-inflammatory cytokine in gout pathology [6,7]. The inflammatory response is characterized by the recruitment of neutrophils and other immune cells, which contribute to the acute inflammatory episodes observed in patients with gout [8].

Moreover, the systemic implications of gout extend beyond joint inflammation, as gout is associated with various comorbid conditions, including renal, cardiovascular, and metabolic disorders. This systemic aspect suggests that intercellular communication plays a crucial role in the pathogenesis of gout, as inflammatory mediators can influence distant organs and potentially exacerbate comorbidities. Recent studies have highlighted the importance of exosomes in mediating intercellular communication, particularly in chronic inflammatory diseases [9]. Exosomes, small membrane-bound vesicles ranging from 30 to 150 nm, are released by various cell types and carry proteins, lipids, and RNA, facilitating the transfer of bioactive molecules between cells [10]. This unique characteristic of exosomes positions them as pivotal mediators of the inflammatory processes associated with gouty arthritis.

The role of exosomes in gouty arthritis is emerging as a significant area of research, as they may contribute to the propagation of inflammatory signals and the modulation of immune responses. For instance, exosome proteins derived from neutrophils and macrophages can influence the behavior of surrounding cells [11], enhancing or dampening inflammatory responses. Furthermore, alterations in the composition of exosome cargo in patients with gout could potentially serve as biomarkers of disease activity and therapeutic response [12,13]. The exploration of exosome-mediated intercellular communication in the context of gouty arthritis not only aids in understanding the underlying mechanisms of the disease but also opens avenues for novel therapeutic strategies targeting these vesicles to modulate inflammation and improve patient prognosis.

In this review, we focus on exosomes, operationally defined as small extracellular vesicles (sEVs) with a size range of ~30–150 nm that are derived from the endosomal pathway and bear typical markers (e.g., CD9, CD63, TSG101) as per the Minimal Information for Studies of Extracellular Vesicles (MISEV) guidelines [14]. While earlier studies may have used the term more broadly, we have critically evaluated the cited literature to ensure that the discussed vesicles align with this definition in the context of gouty arthritis.

**Figure 1:**
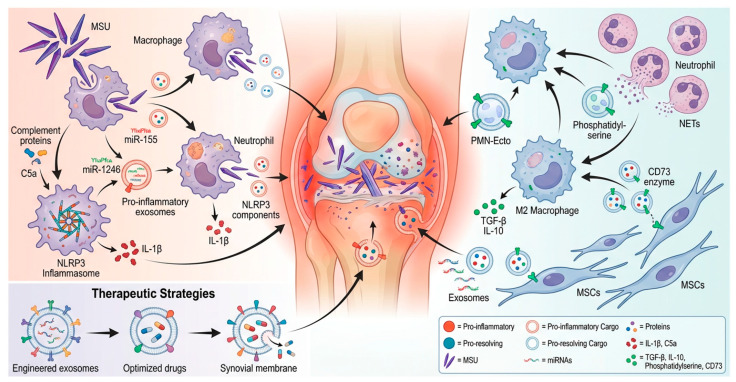
Gouty Arthritis Pathogenesis and Exosome-Mediated Intercellular Communication.

The interplay between MSU crystal deposition, innate immune activation, and exosome-mediated signal amplification is summarized in Figure 1.

This diagram illustrates the dual regulatory role of immune cells (macrophages and neutrophils) and their secreted exosomes in the inflammatory process, along with engineered exosome-based therapeutic strategies. The left panel depicts the interactive network of pro-inflammatory and anti-inflammatory immune cells and their mediators. The right panel presents therapeutic approaches involving engineered exosomes loaded with specific inflammation-related molecules for targeted delivery to affected tissues (e.g., synovial membrane) to achieve precise immune modulation.

## 2. The Biomechanism of Exosomes and Gouty Arthritis

### 2.1. Basic Characteristics and Biological Functions of Exosomes

Exosomes are nanosized extracellular vesicles, typically ranging from 30 to 150 nm in diameter, and play a critical role in intercellular communication [15]. They are secreted by various cell types and contain a diverse array of biomolecules, including proteins, lipids, and nucleic acids, which reflect the physiological state of their parent cells. Exosomes are formed by the inward budding of the endosomal membrane, leading to the formation of multivesicular bodies (MVBs), which subsequently fuse with the plasma membrane to release exosomes into the extracellular space [16]. This process is regulated by various proteins, including Rab guanosine triphosphatases (GTPases), which are crucial for vesicle trafficking and fusion processes [17]. The composition of exosomes can vary significantly depending on the cell type and physiological or pathological conditions, making them valuable tools for diagnostic and therapeutic purposes [18].

#### 2.1.1. Composition and Biological Roles of Exosomes

Exosomes are composed of a lipid bilayer that encapsulates various bioactive molecules, including proteins, lipids, mRNAs, and non-coding RNAs such as microRNAs (miRNAs) [19]. The specific composition of exosomes is determined by the parent cell type and its physiological state, allowing them to serve as indicators of cellular health and disease. For instance, exosomes derived from immune cells can carry immunomodulatory proteins and miRNAs that influence the immune response, whereas those derived from cancer cells may contain oncogenic factors that promote tumorigenesis, such as lncRNAs [20]. The biological functions of exosomes are diverse, ranging from mediating cell-to-cell communication and influencing cellular behavior to acting as vehicles for drug delivery in therapeutic applications [21]. Their ability to transfer functional biomolecules to recipient cells enables them to play significant roles in various physiological and pathological processes, including inflammation [22], tissue repair, and cancer metastasis [23].

#### 2.1.2. Isolation and Characterization of Exosomes

The isolation and characterization of exosomes are critical for understanding their roles in gout and other conditions. Exosomes can be separated from biological fluids, such as plasma, serum, and synovial fluid, using techniques such as ultracentrifugation and size exclusion chromatography [24]. These methods allow for the concentration of exosomes while minimizing contamination by other extracellular vesicles and proteins. Characterization is typically performed using nanoparticle tracking analysis (NTA) [14], which provides information on the size and concentration of exosomes, and electron microscopy [25], which allows the visualization of their morphology. In addition, the presence of specific exosome markers, such as CD9, CD63, and TSG101, was confirmed using Western blotting [26]. These markers are essential for verifying the identity of isolated exosomes and ensuring that they have the characteristics typical of exosomes rather than other types of vesicles.

#### 2.1.3. Biogenesis and Pathological Cargo Loading in Gout

The formation and secretion of exosomes are complex processes that begin with the invagination of the endosomal membrane, leading to the creation of intraluminal vesicles within MVBs. These MVBs can either fuse with lysosomes for degradation or with the plasma membrane to release exosomes into the extracellular environment [27]. The sorting of cargo into exosomes is a highly regulated process influenced by various factors, including cellular context and external stimuli. Key proteins involved in this process include the endosomal sorting complex required for transport (ESCRT) machinery, which facilitates the incorporation of specific proteins and RNAs into budding vesicles [28]. Additionally, Rab GTPases, particularly Rab27, are essential for the transport of MVBs to the cell membrane and their subsequent fusion, ensuring efficient release of exosomes [17]. This mechanism allows exosomes to deliver their cargo to distant cells, influencing various biological processes, including immune modulation and cell-to-cell communication.

In the inflammatory milieu of a gouty joint, exosome biogenesis and cargo loading are significantly altered. The presence of MSU crystals and pro-inflammatory cytokines stimulates cells to release exosomes with distinct characteristics [29]. For instance, plasma exosomes from a rat model of GA were significantly smaller and present in lower concentrations compared to healthy controls, suggesting that the disease state profoundly impacts exosome biogenesis and release dynamics [11].

Recent proteomic and transcriptomic studies have systematically compared exosomal cargo between GA patients and healthy controls, revealing distinct molecular signatures associated with disease activity. Table 1 summarizes key exosomal molecules identified through omics approaches, along with their expression trends. For instance, proteomic analyses of synovial fluid-derived exosomes from GA patients show upregulation of proteins involved in neutrophil degranulation (e.g., S100A8/A9) and lysosomal dysfunction (e.g., Cathepsin D), while enzymes like HPRT1 are downregulated, reflecting impaired purine salvage pathways [30]. Similarly, miRNA sequencing of plasma exosomes has identified a panel of differentially expressed miRNAs, including pro-inflammatory miR-155 and miR-17, which correlate with disease severity and may serve as non-invasive biomarkers [31]. These omics-driven insights not only validate the pathological relevance of exosomal cargo but also highlight potential targets for therapeutic intervention.

### 2.2. The Involvement and Regulatory Mechanisms of Exosomes in the Pathogenesis of Gout

#### 2.2.1. Characteristics of Exosomes and Their Sources in Gout

In the context of gout, exosomes originate from several key cell types, including synovial macrophages, neutrophils, endothelial cells [11], renal tubular cells, hepatocytes, adipocytes, and stromal cells. Each of these cells contributes to the inflammatory milieu characteristic of gout, an inflammatory arthritis caused by monosodium urate crystal deposition in the joints. The involvement of synovial macrophages and neutrophils is particularly significant, as they are the primary responders to inflammation and are involved in the phagocytosis of monosodium urate crystals [8]. Endothelial cells contribute to the vascular aspect of inflammation, whereas renal tubular and hepatocyte-derived exosomes may reflect systemic metabolic responses to hyperuricemia, a common precursor of gouty arthritis [38]. Adipocytes and stromal cells also play roles in the inflammatory response, potentially influencing the secretion profiles of exosomes and their cargo, including proteins and microRNAs, which can modulate inflammation and tissue repair processes [39]. A summary of the major cellular sources of exosomes in GA, along with their key cargo and proposed pathological or therapeutic roles, is provided in Table 2.

#### 2.2.2. Changes in Protein and miRNA Profiles of Exosomes in Gout Models

In gout models, the protein and microRNA (miRNA) profiles of exosomes exhibit significant changes compared to those of healthy controls [13]. These alterations reflect the underlying inflammatory processes and may provide insights into disease progression and potential therapeutic targets for the disease. For instance, studies have identified differentially expressed proteins associated with inflammation and oxidative stress in exosomes derived from patients with gouty arthritis. Similarly, the miRNA content of exosomes can indicate the activation of specific signaling pathways involved in the inflammatory response [31]. Such changes in exosome cargo not only serve as potential biomarkers for the diagnosis and monitoring of gout but also highlight the role of exosomes in mediating communication between cells in the inflammatory environment of gouty arthritis. Identifying specific proteins and miRNAs that are upregulated or downregulated in gout can aid in the development of targeted therapies aimed at modulating the inflammatory response and improving patient outcomes.

#### 2.2.3. The Role of Exosomes in Uric Acid Crystal-Induced Inflammatory Response

The inflammatory response in gouty arthritis is primarily driven by the deposition of MSU crystals in the joints, which activates the innate immune system, particularly through the actions of macrophages and neutrophils [43]. Exosomes released from these activated immune cells have been shown to contain pro-inflammatory cytokines and microRNAs that can propagate inflammatory signals to neighboring cells [44,45]. For instance, exosomes derived from MSU-stimulated neutrophils inhibit osteoblast viability and promote inflammatory responses, thereby contributing to joint damage and pain [46]. Additionally, proteomic analyses have identified that exosomes in the synovial fluid of patients with gout exhibit altered protein expression profiles, including increased levels of inflammatory mediators such as IL-6 and IL-8, which correlate with the severity of inflammation and joint damage [47]. These findings highlight the dual role of exosomes in enhancing the inflammatory response and potentially serving as biomarkers of GA severity.

#### 2.2.4. Activation of Inflammatory and Complement Pathways

Activation of inflammatory and complement pathways is a hallmark of gout. Upon deposition of MSU crystals, the innate immune system is activated, leading to the recruitment of neutrophils and macrophages to the site of inflammation. This response is primarily mediated by the NLRP3 inflammasome, which facilitates the processing and release of pro-inflammatory cytokines, such as IL-1β [48]. Furthermore, the complement system plays a critical role in amplifying inflammatory responses. Complement components, particularly C3a and C5a, are generated during the activation of the complement cascade and contribute to immune cell recruitment and activation. Recent research has indicated that dysregulation of complement activation is associated with increased severity of gout flares [49]. The interplay between the complement system and inflammatory mediators underscores the complexity of gout pathogenesis and highlights potential therapeutic targets for managing acute gouty attacks. Therapeutic strategies aimed at modulating these pathways may offer new avenues for gout treatment.

#### 2.2.5. Lysosomal and Autophagic Dysfunction

Lysosomal and autophagic dysfunction are significant contributors to the pathogenesis of gout [50]. The autophagy-lysosomal pathway is crucial for cellular homeostasis, including the degradation of damaged organelles and proteins. In gout, the accumulation of MSU crystals impairs lysosomal function, leading to decreased autophagy. This impairment not only affects uric acid clearance but also promotes inflammation through the release of pro-inflammatory cytokines. Studies have shown that lysosomal-associated proteins are differentially expressed in the exosomes of patients with gout [11], indicating that lysosomal dysfunction may serve as a biomarker for acute gout flares. Moreover, the activation of the NLRP3 inflammasome by MSU crystals is closely linked to autophagic dysfunction [34], suggesting that restoring autophagic activity may be a potential therapeutic approach for mitigating gouty inflammation. Enhancing lysosomal function and autophagic flux may reduce inflammatory responses and improve the outcomes of patients with gout.

#### 2.2.6. Regulation of Purine and Uric Acid Metabolism

The regulation of purine and uric acid metabolism is pivotal to the pathogenesis of gout. Purines, derived from dietary sources and cellular turnover, are metabolized to uric acid, the accumulation of which can lead to hyperuricemia and subsequent gout [51]. Genetic factors, particularly variations in urate transporter genes, significantly influence uric acid levels [52]. For instance, the ATP-binding cassette transporter ABCG2 plays a critical role in urate excretion, and polymorphisms in this gene are associated with an increased risk of gout [30]. Furthermore, recent studies have identified that dysregulation of the balance between uric acid production and excretion is exacerbated by environmental factors, such as diet, obesity, and alcohol consumption [41]. The interplay between these factors and the resultant increase in uric acid levels can trigger the activation of the NLRP3 inflammasome, which is central to the inflammatory response in patients with gout [48]. Therefore, understanding the mechanisms regulating purine metabolism and uric acid levels is essential for developing effective therapeutic strategies to manage gout.

Exosomes serve as a critical link between systemic metabolic dysfunction and local joint inflammation [53]. Proteomic studies have identified significant alterations in proteins related to purine metabolism within GA-associated exosomes. For example, a significant downregulation of Hypoxanthine-guanine phosphoribosyltransferase 1 (HPRT1), a key enzyme in the purine salvage pathway, was found in the renal tissues of GA model rats [11]. This suggests that exosomes may communicate signals from the inflamed joint back to the kidney, potentially exacerbating the systemic hyperuricemia that drives the disease.

#### 2.2.7. The Impact of Exosomes on Joint Cells and Their Signaling Pathways

Exosomes not only modulate inflammation but also directly affect joint cells, such as chondrocytes and synoviocytes [42]. They can alter the signaling pathways within these cells, influencing processes such as apoptosis, proliferation, and matrix remodeling. For instance, exosomal miRNAs have been implicated in the regulation of chondrocyte function, whereby specific miRNAs can suppress catabolic pathways while promoting anabolic processes in the cartilage [54]. Furthermore, exosomes derived from bone marrow mesenchymal stem cells (MSCs) have shown potential in enhancing cartilage repair and reducing inflammation in animal models of osteoarthritis [55], suggesting a therapeutic avenue for GA. The interaction between exosomes and joint cells underscores their importance in the pathogenesis of gouty arthritis, as they can either exacerbate the inflammatory milieu or promote repair mechanisms depending on their cellular origin and content. Therefore, understanding the precise roles of exosomes in these processes could lead to the development of novel therapeutic strategies aimed at modulating their effects on gouty arthritis.

### 2.3. Exosomes and Their Interaction with Immune Cells

Exosomes, nanosized extracellular vesicles, play a crucial role in intercellular communication and have significant implications for immune responses [56]. Their ability to transfer proteins, lipids, and nucleic acids allows them to influence the behavior of recipient cells, including immune cells, such as macrophages and T cells. These vesicles are secreted by various cell types and carry a diverse array of molecular cargo, including proteins, lipids, and RNAs. This cargo can modulate the behavior of recipient cells, thereby affecting immune activation and inflammation.

#### 2.3.1. Role of Exosomes in Macrophage Activation

Exosomes derived from various cell types have been shown to modulate macrophage activation and function [57]. For instance, exosomes released from mesenchymal stem cells (MSCs) can enhance macrophage polarization towards an anti-inflammatory phenotype (M2), which is beneficial for tissue repair and regeneration [58]. In rheumatoid arthritis (RA), exosomes have been implicated in the regulation of macrophage activity, influencing cytokine production and phagocytosis. Studies have demonstrated that exosomes can carry microRNAs and proteins that alter the inflammatory response of macrophages, promoting a shift from a pro-inflammatory (M1) to an anti-inflammatory (M2) phenotype [59]. This modulation is particularly relevant in chronic inflammatory conditions, where macrophages play a pivotal role in sustaining the inflammation and tissue damage. Moreover, exosomes can serve as carriers of disease-specific biomarkers, allowing the identification of macrophage activation states in various diseases [33]. The therapeutic potential of exosomes is further underscored by their ability to deliver anti-inflammatory agents directly to macrophages, thereby enhancing their efficacy in the treatment of inflammatory diseases.

#### 2.3.2. Regulation of T Cell Function by Exosomes

Exosomes also play a critical role in modulating T cell function by influencing their activation, proliferation, and differentiation [60]. For instance, exosomes derived from tumor cells can carry immunosuppressive factors that inhibit T cell responses, contributing to tumor immune evasion. In various cancers, tumor-derived exosomes have been shown to induce T cell exhaustion by upregulating inhibitory receptors, such as PD-1 and TIM-3, on T cells, thereby impairing their cytotoxic functions [61]. This mechanism highlights the dual role of exosomes as mediators of intercellular communication and as potential therapeutic targets in cancer immunotherapy. Additionally, exosomes derived from activated T cells enhance the immune response by transferring functional proteins and RNAs that promote T cell activation and proliferation [62]. For example, exosomal miRNAs have been identified as key regulators of T cell differentiation and function, influencing the pathways involved in T cell activation and memory formation [63]. The ability of exosomes to modulate T cell responses offers exciting opportunities for therapeutic interventions, particularly to enhance antitumor immunity and restore T cell function in patients with autoimmune diseases.

#### 2.3.3. How Exosomes Affect the Activation of Immune Cells and Cytokine Production

Exosomes are key players in immune cell activation and modulation of cytokine production. They can deliver specific miRNAs and proteins that influence the signaling pathways within recipient immune cells, such as T cells, B cells, and macrophages. For example, exosomal miRNAs can regulate the expression of cytokines by targeting messenger RNAs (mRNAs) of these cytokines, effectively altering the immune landscape [19]. Studies have shown that exosomes derived from dendritic cells can enhance T cell activation by presenting antigens and co-stimulatory signals, whereas exosomes from regulatory T cells can suppress the activation of effector T cells and promote tolerance [62]. Furthermore, exosomes can carry inflammatory cytokines that further propagate the inflammatory response in recipient cells. This dual role of exosomes in promoting and inhibiting immune responses underscores their complexity and potential as therapeutic targets for immunological disorders.

#### 2.3.4. The Role of Exosomes in Regulating Macrophage and Neutrophil Responses

Exosomes play a pivotal role in modulating the responses of macrophages and neutrophils [64], which are critical components of innate immunity. Macrophages secrete exosomes containing pro-inflammatory cytokines and miRNAs, which enhance the inflammatory response and promote the differentiation of other immune cells [65]. For instance, exosomes derived from M1 macrophages (classically activated macrophages) can stimulate the polarization of naïve macrophages towards a pro-inflammatory phenotype, thereby amplifying the immune response during infection or tissue injury [66]. In contrast, exosomes from M2 macrophages (alternatively activated macrophages) can promote tissue repair and resolution of inflammation by delivering anti-inflammatory signals and promoting the polarization of other macrophages towards a healing phenotype [58]. Neutrophils, which are known for their rapid response to infection, also interact with exosomes. Studies have demonstrated that neutrophil-derived exosomes can influence macrophage behavior and promote inflammation by releasing pro-inflammatory mediators [67]. Conversely, macrophage-derived exosomes can modulate neutrophil activity by enhancing their ability to respond to pathogens or by promoting apoptosis to resolve inflammation [6,68]. This intricate interplay between exosomes, macrophages, and neutrophils illustrates the importance of exosomes in orchestrating immune responses and maintaining immune system homeostasis.

### 2.4. The Potential Impact of Exosomes in the Progression of Gouty Arthritis

Exosomes, small extracellular vesicles secreted by various cell types, play a significant role in the pathophysiology of gouty arthritis. Recent studies have highlighted their role in mediating intercellular communication and influencing inflammatory processes associated with GA [69]. Exosomes carry various biomolecules, including proteins, lipids, and nucleic acids, which reflect the physiological state of their parent cells. In the context of GA, exosomes have been shown to contain specific miRNAs and proteins involved in regulating inflammation and immune responses, suggesting their potential utility as biomarkers for disease progression and therapeutic targets. Characterization of exosome contents from the synovial fluid of patients with GA revealed distinct profiles that correlated with disease severity, indicating that exosomes could serve as noninvasive diagnostic tools for monitoring disease activity and treatment response [30].

#### 2.4.1. The Prospective Application of Exosomes as Biomarkers

Exosomes hold great promise as biomarkers for gouty arthritis due to their stability in biological fluids and their ability to encapsulate and transport diverse biomolecules. Research has identified specific exosomal miRNAs and proteins that are differentially expressed in GA patients compared to healthy controls, suggesting their potential as diagnostic markers [31]. The integration of proteomic and metabolomic analyses with exosome profiling can provide insights into the underlying mechanisms of GA and aid in identifying novel therapeutic targets. For instance, urinary exosomes from GA patients exhibit altered levels of proteins involved in lysosomal function (e.g., Cathepsin D) and ferroptosis (e.g., ACSL4, VDAC2), which may serve as indicators of acute gout flares [12,13,70]. Similarly, distinct signatures of exosomal miRNAs in plasma, such as elevated miR-155 and miR-17 alongside altered miR-223 levels, correlate with disease severity [35]. The feasibility of isolating exosomes from accessible biofluids like blood and synovial fluid enhances their clinical utility [24], paving the way for non-invasive monitoring of disease progression and therapeutic efficacy in GA.

#### 2.4.2. The Potential Applications of Exosomes in Therapy

Exosomes are promising biomarkers and therapeutic agents for GA treatment. Their natural ability to transport bioactive molecules makes them ideal candidates for targeted drug delivery. Recent advancements have focused on engineering exosomes to enhance their therapeutic potential, such as loading them with anti-inflammatory agents or uricase to directly address elevated uric acid levels, which are characteristic of GA [71]. Moreover, exosomes derived from M2 macrophages have been shown to possess anti-inflammatory properties [6], suggesting that they can be used to effectively modulate the immune response in GA. The development of exosome-based therapies could lead to innovative treatments that not only alleviate symptoms but also target the underlying mechanisms of the disease. Furthermore, the incorporation of exosomes in combination therapies could enhance the efficacy of existing treatments, providing a multifaceted approach to GA management. Overall, the therapeutic applications of exosomes in GA present a novel avenue for research and clinical practice, with the potential to significantly improve patient outcomes.

### 2.5. The Relationship Between Exosomes and Drug and Herbal Treatments

Exosomes, small extracellular vesicles that facilitate intercellular communication, have gained attention for their roles in drug delivery and therapeutic applications in recent years. Chemical drugs can modulate exosome secretion, cargo composition, and uptake to exert therapeutic effects. For instance, studies have shown that certain chemotherapeutic agents can influence the release of exosomes from cancer cells, which carry therapeutic biomolecules that enhance drug efficacy [72]. This is particularly relevant in the context of cancer treatment, where exosomes can transport chemotherapeutic agents or RNA molecules that can modify the tumor microenvironment, thereby improving drug delivery and reducing side effects. For example, exosomes derived from mesenchymal stem cells (MSCs) have been shown to possess immunomodulatory properties and promote tissue repair, making them promising candidates for targeted drug delivery systems. Furthermore, exosome characterization can provide insights into the mechanisms by which drugs exert their effects, as exosomal content can reflect the metabolic state of the cells from which they originate [36].

Herbal components also significantly influence exosome-mediated signaling pathways, thereby enhancing therapeutic outcomes [73]. Various studies have shown that herbal extracts can alter the composition and function of exosomes, thereby modulating their effects on target cells [74]. For instance, certain herbal medicines have been shown to enhance the release of exosomes containing microRNAs that regulate the gene expression involved in inflammation and cancer progression. In the context of gouty arthritis, herbal treatments have been found to affect exosome-mediated pathways, suggesting that these treatments can modulate the inflammatory response by regulating the exosome cargo. Additionally, herbal-derived exosome-like nanovesicles have emerged as a novel approach in cancer therapy, exhibiting therapeutic benefits by directly delivering bioactive compounds to target cells. This highlights the potential of combining herbal treatments with exosome technology to develop more effective therapeutic strategies that leverage the natural properties of exosomes and herbal compounds.

In conclusion, the relationship between exosomes and both chemical drugs and herbal treatments is a burgeoning area of research that requires further investigation. Understanding how chemical agents and herbal compounds influence exosome dynamics can lead to innovative therapeutic strategies that enhance drug delivery and efficacy, while minimizing adverse effects. This integrated approach may pave the way for the development of more effective and personalized treatment modalities for various diseases, particularly cancer and inflammation.

### 2.6. Therapeutic Potential of Exosomes in Gouty Arthritis

Exosome research has witnessed significant advancements in recent years, particularly in understanding the role of exosomes in various diseases and their therapeutic potential [75]. Exosomes, small extracellular vesicles, are involved in intercellular communication and carry a diverse array of biomolecules, including proteins, lipids, and RNAs. Recent studies have highlighted the potential of exosomes as biomarkers for diseases such as cancer, inflammatory conditions, and neurodegenerative disorders, as well as their utility in drug delivery systems [21]. As research progresses, the focus is shifting towards harnessing exosomes for therapeutic applications, including their use in regenerative medicine and targeted therapies.

#### 2.6.1. Engineered Exosomes for Targeted Drug Delivery

Exosome-related therapeutic strategies are emerging as novel approaches for various diseases, demonstrating significant potential in promoting tissue regeneration and repair in conditions such as diabetic wounds, spinal cord injury, and neurodegenerative diseases. As a near-ideal natural drug delivery system, exosomes offer superior biocompatibility, low immunogenicity, and the ability to cross biological barriers, making them advantageous over many synthetic nanoparticles [21]. Engineered exosomes further enhance these capabilities through modifications that improve stability and targeting precision [76]. In the context of gouty arthritis (GA), several strategies are being explored: loading exosomes with conventional anti-inflammatory agents like colchicine or corticosteroids, or with biologics such as IL-1 receptor antagonists (IL-1Ra) to achieve localized anti-inflammatory effects [77]; encapsulating urate-lowering enzymes like uricase to create a targeted “urate sink” within the joint; and engineering exosome surfaces with specific ligands (e.g., antibodies or peptides) to enhance binding to receptors highly expressed on inflamed synovial tissues or activated immune cells [78]. Beyond GA, the ability of exosomes to cross barriers such as the blood–brain barrier underscores their broader therapeutic potential for central nervous system disorders. However, challenges remain in standardizing production, ensuring consistent therapeutic outcomes, and addressing safety concerns. Future research will likely focus on optimizing exosome engineering techniques, exploring their role in combination therapies, and validating their efficacy through clinical trials across diverse therapeutic contexts.

#### 2.6.2. MSC-Derived Exosomes as Immunomodulatory Agents

Mesenchymal stem cell-derived exosomes (MSC-exos) represent a promising cell-free therapeutic strategy for GA due to their intrinsic anti-inflammatory and tissue-repair properties [79]. Unlike exosomes derived from inflamed joint cells, MSC-exos carry a distinct cargo enriched in anti-inflammatory miRNAs (e.g., miR-223, miR-146a), cytokines (e.g., TGF-β, IL-10) [80], and enzymes that modulate immune responses. In experimental GA models, MSC-exos have been shown to suppress NLRP3 inflammasome activation, promote M2 macrophage polarization, reduce neutrophil infiltration, and attenuate cartilage degradation. These effects are mediated through the transfer of bioactive molecules that reprogram immune cells and restore joint homeostasis. Importantly, MSC-exos circumvent the safety concerns associated with whole-cell therapies, such as tumorigenicity and immunogenicity, making them attractive candidates for clinical translation. Ongoing research is focused on optimizing exosome isolation protocols, enhancing targeting efficiency [81], and developing scalable production methods to facilitate their application in GA management [82].

#### 2.6.3. Herbal Compounds and Exosome Modulation in GA Therapy

Traditional herbal medicines have long been used in the management of gout and inflammatory arthritis. Emerging evidence suggests that certain herbal extracts can modulate exosome biogenesis, cargo loading, and recipient cell responses, thereby exerting anti-inflammatory and immunomodulatory effects [83]. For instance, bioactive compounds such as berberine (from Coptis chinensis), curcumin (from Curcuma longa), and paeoniflorin (from Paeonia lactiflora) have been reported to alter the miRNA and protein profiles of exosomes released from macrophages and synoviocytes, favoring an anti-inflammatory phenotype. Additionally, herbal-derived exosome-like nanovesicles (ELNs) have been investigated as natural drug carriers capable of delivering phytochemicals directly to inflamed joints [84]. These ELNs exhibit low immunogenicity and high biocompatibility, offering a novel avenue for integrative GA therapy. Further research is needed to elucidate the specific mechanisms by which herbal components influence exosome-mediated communication and to validate their efficacy in clinical settings.

#### 2.6.4. Challenges and Future Directions

Despite the promising advances in exosome research, several challenges and opportunities remain in this field. One of the primary challenges is the standardization of exosome isolation and characterization methods, which can significantly affect the reproducibility and reliability of the research findings. Additionally, the biological variability of exosomes derived from different cell types poses a challenge to their clinical application. Comprehensive studies are needed to elucidate the mechanisms by which exosomes exert their effects in various biological contexts, particularly in disease progression and therapeutic response. However, these challenges present opportunities for innovation in the field of exosome research. The development of novel isolation techniques, such as microfluidic devices and nanotechnology-based approaches, may enhance exosome yield and purity. Furthermore, interdisciplinary collaboration among researchers, clinicians, and industry stakeholders can facilitate the translation of exosome-based therapies from bench to bedside [33]. As the field continues to evolve, addressing these challenges will be crucial for unlocking the full potential of exosomes in medicine and improving patient outcomes.

The potential applications of exosomes in the clinical diagnosis and treatment of gout have gained attention in the medical field. Exosomes, nanoscale extracellular vesicles, can carry various biomolecules, including proteins, lipids, and nucleic acids, making them promising candidates for non-invasive diagnostic tools. Recent studies have indicated that specific proteins associated with lysosomal function and ferroptosis are differentially expressed in the urinary exosomes of patients with gouty arthritis. For instance, proteins such as Cathepsin Z (CTSZ) and AP-1 complex subunit beta-1 (AP1B1) have shown significant predictive value for acute gout attacks, with areas under the curve (AUC) exceeding 0.8 in receiver operating characteristic (ROC) analyses [12]. This suggests that exosomal proteins could serve as biomarkers for diagnosing acute gout and monitoring disease progression and response to therapy. Future research should focus on validating these findings in larger cohorts and exploring the mechanistic roles of these proteins in the pathophysiology of gout. Additionally, the integration of exosome analysis into routine clinical practice could enhance the accuracy and efficiency of gout diagnosis and management.

Further exploration of exosomes as potential biomarkers is warranted, particularly for gout. Exosomes reflect the physiological and pathological states of their parent cells, providing insights into disease mechanisms. For example, the identification of specific miRNAs within exosomes has been proposed as a novel approach for monitoring disease activity and therapeutic responses in various conditions, including gouty arthritis [85]. The stability and resistance to degradation of exosomal miRNAs make them ideal candidates for biomarker development. Future research should aim to identify miRNA signatures associated with gout and assess their clinical utility [86]. Furthermore, the development of standardized protocols for exosome isolation and characterization is crucial to advance this field of research. By leveraging the unique properties of exosomes, researchers can potentially uncover new biomarkers that can revolutionize the diagnosis and treatment of gout, leading to more personalized and effective therapeutic strategies.

## 3. Conclusions

The exploration of exosomes in the pathogenesis of gouty arthritis has unveiled a crucial dimension of intercellular communication that significantly influences the inflammatory processes. While exosomes play a central role in propagating inflammation in GA, they also represent promising therapeutic targets and vehicles for drug delivery. Notably, MSC-derived exosomes and herbal-modulated exosomes offer novel strategies for immunomodulation and inflammation resolution, distinct from conventional anti-inflammatory drugs. This review highlights the multifaceted roles of exosomes, not only as mediators of cellular communication but also as potential biomarkers and therapeutic targets for the management of gout. As our understanding of the molecular mechanisms underlying gout continues to evolve, it has become evident that exosomes serve as vital conduits for the transfer of inflammatory mediators, genetic material, and signaling molecules among cells in the affected joints.

The role of exosomes in the pathogenesis of gouty arthritis is a pivotal advancement in our understanding of this complex disease. Exosomes, small extracellular vesicles, play an integral role in mediating inflammatory responses and modulating immune cell function. Their capacity to transport and transfer a diverse array of bioactive molecules, including proteins, lipids, and nucleic acids, underscores their potential as key mediators of the inflammatory cascade characteristic of gouty arthritis. The interaction between exosomes and immune cells suggests that these vesicles influence disease progression by regulating inflammatory pathways.

From an expert perspective, it is essential to balance the various research findings on exosomes in patients with gout. While current studies have established the involvement of exosomes in the pathophysiology of this disease, further investigation is warranted to delineate their exact roles and mechanisms of action. This includes understanding the diverse populations of exosomes derived from different cell types and their specific contributions to the inflammatory milieu characteristic of gouty arthritis. Moreover, the interplay between exosome content and immune response in gouty arthritis presents a complex landscape that requires careful analysis to avoid oversimplification of the results.

Exosomes represent a promising therapeutic target for the management of gout. By harnessing these capabilities, we can develop innovative strategies to mitigate inflammation and modify disease progression. This necessitates a concerted effort from both basic researchers and clinicians to translate these findings into clinical practice. Future investigations should prioritize elucidating the specific mechanisms through which exosomes contribute to gout pathophysiology, with particular attention to their potential as diagnostic biomarkers. The identification of exosomal biomarkers could facilitate the development of novel diagnostic tools and targeted therapeutic strategies, ultimately enhancing patient outcomes. Additionally, exploring exosome-based therapeutic approaches offers promising avenues for modulating inflammatory responses and restoring immune homeostasis in patients with IBD.

Achieving these objectives necessitates a collaborative interdisciplinary approach involving researchers, clinicians, and pharmaceutical developers. Such collaboration is essential to bridge the gap between laboratory discoveries and clinical applications, ensuring that insights into exosome function translate into tangible benefits for patients with cancer. In conclusion, the integration of exosome research into the broader context of gouty arthritis opens new avenues for understanding and treating this disease. Balancing the various research perspectives on exosomes will be pivotal in developing a comprehensive approach to gout management, ultimately improving patient outcomes. The journey towards leveraging exosomes in clinical practice is just beginning, and the promise they hold underscores the importance of continued research in this dynamic field. Ongoing research on exosomes in gouty arthritis holds significant promise for unveiling innovative therapeutic options, improving disease management, and enhancing patient prognosis.

## Figures and Tables

**Table 1:** Key Exosomal Cargo Molecules Identified in Gouty Arthritis and Their Pathological Roles.

Cargo Type	Key Molecules	Pathological Role in Gout	Expression Trend in GA	Key References
Proteins	S100A8/A9, Lysozyme C	Associated with neutrophil degranulation and activation	↑	[32]
	Cathepsin D (CTSD)	Upregulated; indicates lysosomal dysfunction	↑	[11]
	HPRT1	Downregulated; links exosomes to purine metabolism dysregulation	↓	[11]
	Complement Components	C3a, C5a; amplify inflammatory responses	↑	[33]
	NLRP3 Inflammasome Components	Can be transferred to recipient cells to activate NF-κB	↑	[30,34]
miRNAs	miR-155, miR-17, miR-18a	Upregulated; promote pro-inflammatory macrophage polarization	↑	[35]
	miR-1246	Transferred from neutrophils to inhibit osteoblast function, promoting bone erosion	↑	[36]
	miR-223	Paradoxically upregulated; may be a compensatory anti-inflammatory signal	↑ (paradoxical)	[35]
Lipids	Phosphatidylserine (PS)	Exposed on pro-resolving microvesicles; engages MerTK receptor	↑	[37]

**Table 2:** Sources of Exosomes in Gouty Arthritis and Their Proposed Roles.

Cell Source	Exosome Cargo/Characteristics	Proposed Role in GA	Key References
Synovial Macrophages	Pro-inflammatory cytokines, NLRP3 components	Amplify inflammation, promote neutrophil recruitment	[34]
Neutrophils	miR-1246, pro-resolving lipids (e.g., PS)	Inhibit osteoblast function, modulate resolution	[37]
MSCs	Anti-inflammatory miRNAs, tissue-repair factors	Potential immunomodulation, tissue protection	[21]
Renal Tubular Cells	Proteins linked to purine metabolism (e.g., HPRT1)	Communicate systemic metabolic dysregulation	[12]
Hepatocytes	Xanthine oxidase, acute-phase proteins	Modulate systemic inflammation	[40]
Adipocytes	Adipokines, inflammatory miRNAs	Link metabolic syndrome to GA	[41]
Chondrocytes	Cartilage-degrading enzymes	Contribute to joint damage	[42]

## Data Availability

No new data were created or analyzed in this study. Data sharing is not applicable to this article.

## Abbreviations

The following abbreviations are used in this manuscript:

GA	Gouty arthritis
MSU	monosodium urate
NLRP3	nucleotide-binding oligomerization domain-, leucine-rich repeat-, and pyrin domain-containing protein 3
IL-1β	interleukin-1β
MVBs	multivesicular bodies
GTPases	guanosine triphosphatases
miRNAs	microRNAs
MSCs	mesenchymal stem cells
HPRT1	Hypoxanthine-guanine phosphoribosyltransferase 1
